# Non-specific symptoms and signs of cancer: different organisations of a cancer patient pathway in Denmark

**DOI:** 10.1080/02813432.2021.1880094

**Published:** 2021-02-25

**Authors:** Christina Sadolin Damhus, Volkert Siersma, Susanne Oksbjerg Dalton, John Brodersen

**Affiliations:** a The Research Unit for General Practice and Section of General Practice, Department of Public Health, University of Copenhagen, Copenhagen, Denmark; b The Primary Health Care Research Unit, Region Zealand, Denmark; c Survivorship and Inequality in Cancer, The Danish Cancer Society Research Center, Copenhagen, Denmark; d Department of Clinical Oncology and Palliative Care, Zealand University Hospital, Naestved, Denmark

**Keywords:** Non-specific symptoms, cancer patient pathway, diagnostic work-up, primary healthcare, diagnostic units, questionnaire

## Abstract

**Objective:**

We aimed to investigate the Non-specific Symptoms and Signs of Cancer-Cancer Patient Pathway (NSSC-CPP) in order to describe organisational and clinical practice similarities and differences in the diagnostic work-up of suspected cancer in Denmark.

**Material and methods:**

A questionnaire on the organisation and practice pertaining to the NSSC-CPP was completed by all 21 diagnostic units in the five healthcare regions in Denmark.

**Results:**

The questionnaire responses revealed regional and intraregional differences in the organisation and clinical practice of the NSSC-CPP. CT scan was the most often used imaging in the NSSC-CPP but there was no consensus whether the CT scan should be ordered and evaluated by general practitioners (GPs) or by the diagnostic units. Two regions were consistent but had different modalities regarding referrals from GPs. Three regions had intra-regional differences. The units reported on different types and frequency of forum for patient plan discussion and how to end a NSSC-CPP.

**Conclusion:**

The NSSC-CPP is implemented with great regional and intra-regional differences in Denmark. GPs face different requirements when referring to the NSSC-CPP, which indicates that the division of role and responsibility between GPs and the diagnostic units is not well defined.

**KEY POINTS**
In Denmark, the cancer patient pathway for non-specific symptoms and signs of cancer (NSSC-CPP) has been implemented with variations, but little is known about these different modalities. This study showed that both at a regional and an intra-regional level:
•General practitioners meet different implementation of national guidelines in the diagnostic units when referring to the NSSCP-CPP
•The suitable patient group for the NSSC-CPP is not well defined
•Quality criteria are needed to monitor, evaluate and improve the diagnostic work-up for patients with non-specific symptoms and signs of cancer

## Background

Internationally, diagnosing cancer as early as possible is a priority for the general public and ranks high on the political agenda. It is assumed that a prolonged diagnostic interval—the time between the first presentation of symptoms to a clinician to the time of diagnosis—leads to poorer cancer outcomes [1]. Epidemiological evidence on the association between diagnostic intervals and cancer outcomes is ambivalent, and suggests that the benefit of shortened diagnostic intervals varies by cancer type [2–4]. Still, political attempts to expedite cancer diagnoses have resulted in the implementation of international and national cancer guidelines. In several countries, including Denmark, cancer patient pathways (CPPs) have been implemented. In Denmark, the CPPs should ensure faster diagnosis and, for those patients diagnosed with cancer, result in a rapid initiation of treatment through streamlined, standardised pathways with recommended time frames [5]. The quality of CPPs is often monitored using time frames and CPPs are considered successful as waiting times have significantly reduced across almost all cancer types [6]. Still, the introduction of CPPs should be seen in the context of the expansion of radiotherapy capacity, better imaging equipment and an overall improvement in the treatment of cancer in the same time period [7].

In Denmark, GPs are expected to react to alarm symptoms of cancer as GPs are the gatekeepers to the secondary healthcare system. GPs are challenged by the fact that approximately 15% had experienced alarm symptoms of cancer in a given year [8] while less than 1% of the Danish population is diagnosed with cancer in a given year [9]. Therefore, even when alarm symptoms are identified, the positive predictive value (PPV) of having cancer is low [10,11]. Studies show that the CPPs favour patients presenting with specific alarm symptoms of cancer [12]. Non-specific symptoms have a lower PPV than alarm symptoms and are therefore more difficult for the patient and the GP to recognize [13,14]. Examples of non-specific symptoms are: general feeling of illness, fatigue, pain, weight loss or fever.

To expedite a cancer diagnosis for these patients, the CPP for Non-specific Symptoms and Signs of Cancer (NSSC-CPP) was introduced and implemented in Denmark in 2011–2012 [15]. The aim of the Danish NSSC-CPP was to provide GPs with new referral possibilities when consulting with patients in whom they have a suspicion of serious disease, but who do not fit into the existing CPPs. According to the Danish guidelines, the NSSC-CPP consists initial of two steps [15]. *Step 1* includes patient anamnesis, clinical investigation and blood and urine tests. Thereafter, if continued suspicion of disease, *Step 2* consists of either X-ray and ultrasound of abdomen or CT scan of thorax, abdomen and pelvis [15]. These tests should be ordered by the patient’s GP and the test results should include a conclusion and a guide on the clinical consequences from the radiological department, enabling the GP to make further decisions on possible investigations or treatment plan. If Step 1 *and* Step 2 indicate no obvious explanation, but the GP still has a suspicion of serious disease, the GP is advised to refer the patient to a diagnostic unit which continues the diagnostic work-up. A diagnostic unit is a hospital-based medical centre with comprehensive facilities for medical investigation, including easy access to expertise across a wide range of relevant medical specialties. In Denmark, the secondary healthcare system, including the diagnostic units, is run by the five health regions, and the Danish Health Data Authority (DHDA) reports wide variation in the number of pathways completed across regions [16]. In 2018, this ranged from 390 completed pathways per 100,000 citizens in the Central Region, compared to 96 per 100,000 in Region Zealand [16]. The DHDA concludes that this variation might be due to each region’s choice of local organisation of the pathway. A recent report [17] found that the diagnostic units varied in a number of ways concerning factors such as: their organisational association at the hospital, staff composition, handling of other functions besides NSSC-CPP and use of investigation methods. Unfortunately, the report did only conduct interviews with 10 of the 21 diagnostic units in Denmark. Therefore, no studies have examined the NSSC-CPP from a nationwide perspective taking all the diagnostic units and modalities into consideration. The aim of this study, therefore, was to investigate the NSSC-CPP in order to describe organisational and clinical practice similarities and differences in the diagnostic work-up of suspected cancer in Denmark.

## Material and methods

We developed an online questionnaire to gain insight into the diagnostic units’ organisation and everyday practice. It was constructed based on published scientific literature, national and local guidelines for the Danish NSSC-CPP as well as the first author’s visits in 6 of the 21 diagnostic units in Denmark [6,15,17–19]. The questionnaire was pilot tested for comprehension and usefulness in a focus group including a sample of 40 administrative workers, health professionals and researchers all working with CPPs and attending a meeting about NSSC-CPP in Denmark. A modified version of the think-aloud test was conducted in the focus group [20]. First, all participants were asked to complete a paper version of the questionnaire. Second, one of the authors read each item aloud, and participants were encouraged to comment on the content relevance and the wording of the items. The focus group session lasted approximately 1 h, it was audio-recorded and comments and reflections during the session were taken. In addition, the questionnaire was answered by four health professionals, all working in diagnostic units, which also contributed to the final version of the questionnaire. Based on the focus group and interviews, the questionnaire was revised and items were added. Health professionals from all five health regions in Denmark contributed to the questionnaire development. The questionnaire took approximately 15 min to complete (To view a full version of the questionnaire, see Supplementary Material 1).

The final questionnaire encompassed 26 items with a minimum of 19 items to complete, depending on the answers to specific questions. The questionnaire was sent by E-mail in May 2019 to one designated representative from each of the 21 diagnostic units in Denmark. The representatives (doctors, nurses or medical secretaries) were chosen by the first author based on their knowledge of the clinical aspects of NSSC-CPPs. In some situations, in correspondence with the authors, the representative recommended other health professionals working in the unit to answer the questionnaire. The first author analysing the data had no relation to the participants before the questionnaire development. In Denmark, approval from the Research Ethics Committee is only required for interventional studies.

## Results

Telephone and E-mail reminders were sent in June and July 2019, and we reached 100% completion in August 2019. Most of the responders were medical doctors (90%) and males (62%). The items cover six themes which are presented below. Table 1 offers further details to support the results section and provides colour codes to match items and themes.

**Table 1.** Quantitative results from questionnaire.

N	Item content	Respond Numbers = units if nothing else stated	
1	Number of health professionals in the unit per day. Range (mean)	Secretaries	0,5–5(1.8)
		Medical doctors	1–9(2.3)
		Nurses	0–5(1.8)
2	Staff’s affiliation to the diagnostic unit. E.g. working full time/part time.	Free-text item, summary in the result section	
3	Medical doctors’ education in the units. (more units report more specialities)	Cardiology	1
		Family medicine	9
		Oncology	1
		geriatric	5
		Respiratory	5
		Haematology	6
		Infection medicine	7
		Nephrology	7
		Gastroenterology	8
		Endocrinology	8
		Specialist in training (introductory education)	6
		Specialist in training (main education)	8
4	Organisational affiliation.	Internal medicine	17
		Emergency	2
		Respiratory and infection medicine	1
		Endocrinology	1
5	Is the diagnostic unit located the same place as it’s organizational affiliation?	Yes	17
		No	4
6	Describe how the physical location and the organisational affiliation differ.	Free-text item. Summary in result section	
7	Capacity, number NSSC-CPP referrals accepted in unit.		5-150(49.5)
8	Patients beside NSSC-CPP.	Yes	19
		No	2
9	Other patients than NSSC-CPP.	Patients with cancer of unknown primary site (CUP)	18
		Patients with non-specific symptoms that do not fit into the NSSC-CPP or other organ specific pathways	14
10	Referrals to NSSC-CPP from general practice (GP). Are there information or investigations that needs to be done before referral?	Yes	17
		No	4
11	Process if the necessary information or investigations are not done before referral from GP.	The unit reject the referral and inform the GP about it. Furthermore, the GP can always contact the diagnostic unit	13
		The unit does not reject the referral but gather the necessary information and take the tests themselves	1
		Other: It differ from patient to patient	1
		Other: the unit rejects referral but inform the GP about the reason for rejection	2
12	Required information and investigations before acceptance of referral to NSSC-CPP from GP.	Blood sample	15
		X-ray	4
		Ultrasound	2
		CT scan	8
		Relevant patient information e.g. telephone number	11
		The patient needs to be informed about the initiation of a cancer patient pathway	12
13	Referrals to NSSC-CPP from hospital. Are there information or investigations that needs to be done before referral?	Yes	11
		No	11
14	Process if the necessary information or investigations are not done before referral from hospital.	The unit reject the referral and inform the hospital department about it	1
		The unit reject the referral and inform the GP about it. Furthermore, the GP can always contact the diagnostic unit	8
		The unit does not reject the referral but gather the necessary information and take the tests themselves	1
		Other: It differ from patient to patient	1
15	Required information and investigations before acceptance of referral to NSSC-CPP from hospital.	Blood sample	7
		X-ray	1
		Ultrasound	0
		CT scan	5
		Relevant patient information e.g. telephone number	8
		The patient needs to be informed about the initiation of a cancer patient pathway	10
16	Redirected and rejected referrals %.	Redirected	1–15(6.6)%
		Rejected	0–20(8.1)%
17	Reasons for rejecting referrals to NSSC-CPP.	See Figure 1	
18	Does the patient’s age influence the choice of CT scan as imaging?	Yes	10
		No	11
19	Enter the threshold of age to CT scan.	Free-text item. Summary in results section	
20	Which images do your unit use in the most NSSC-CPP?	X-ray thorax + ultrasound abdomen	1
		CT scan (depend on age)	20
21	Conference use.	See Table 2	
22	Departments attending MDTs.	Free-text item. Summary in result section	
23	Units able to continue diagnostic work-up when cancer is not confirmed.	Yes	10
		No	11
24	The 3 most common hospital departments referring to when a NSSC-CPP is ended.	Gastroenterology(5), Infection medicine(3), Endocrinology(2)	
25	Average calendar days for NSSC-CPP in unit.		5–30(16.8) days
26	Feel free to write all your comments or questions here.	Free-text item. See results section

- The organisation within the diagnostic units.
- Referral requirements when GPs or hospital departments refer to a diagnostic unit.
- Use of imaging in the diagnostic units and the influence of patient age on the use of imaging.
- Redirected and rejected referrals and reasons for rejections.
- Conferences for discussion of patient plans.
- Continuation of diagnostic work-up when cancer is not confirmed.

### The organisation within the diagnostic units

In four units (19%), the physical location and the affiliation differed: in two units the diagnostic work-up of NSSC-CPP was shared between more hospitals; the two remaining units were physical located with other small specialities. Healthcare professionals often divided their time between departments and did not work full-time in the diagnostic units. In some diagnostic units, patients first consulted a nurse who was responsible for the coordination and booking of tests during the pathway. In two units, however, no nurses were employed, and medical secretaries and doctors coordinated the diagnostic pathways.

### Referral requirements when GPs or hospital departments refer to a diagnostic unit

Seventeen units (81%) required GPs to provide information or perform tests before referral; while 11 units (52%) had requirements when the referral came from another hospital department. The respondent from one of the diagnostic units added that they would rather order blood tests themselves to ensure the right blood panel. In eight of the diagnostic units (38%), GPs were required to order a CT scan before the referral was accepted. Referral criteria were consistent across two regions, and inconsistent across three regions. One region, across all of its diagnostic units, required GPs in all cases to perform blood tests before referral and to inform patients about the initiation of the NSSC-CPP. The other consistent region required, in addition, that a CT scan was ordered before referral to a diagnostic unit. Within each of the remaining three regions, some diagnostic units had no requirements of GPs and others required diagnostic work-up including CT scan before referral.

### Use of imaging in the diagnostic units and the influence of patient age on the use of imaging

All but one of the diagnostic units (95%) reported that a CT scan was the standard investigative method used in most NSSC-CPPs. In the free-text option in the questionnaire, one respondent wrote that the unit used low-dose CT scans and another unit added a CT scan of the pelvis. Also, one diagnostic unit reported that a ‘safety CT’ was performed in most of their NSSC-CPPs, to ensure that nothing was missed. Ten units reported that patients younger than 35–40 years were not automatically CT scanned. These units did not have a specific threshold but each patient’s case was evaluated.

### Redirected and rejected referrals and reasons for rejections

**Figure 1.**
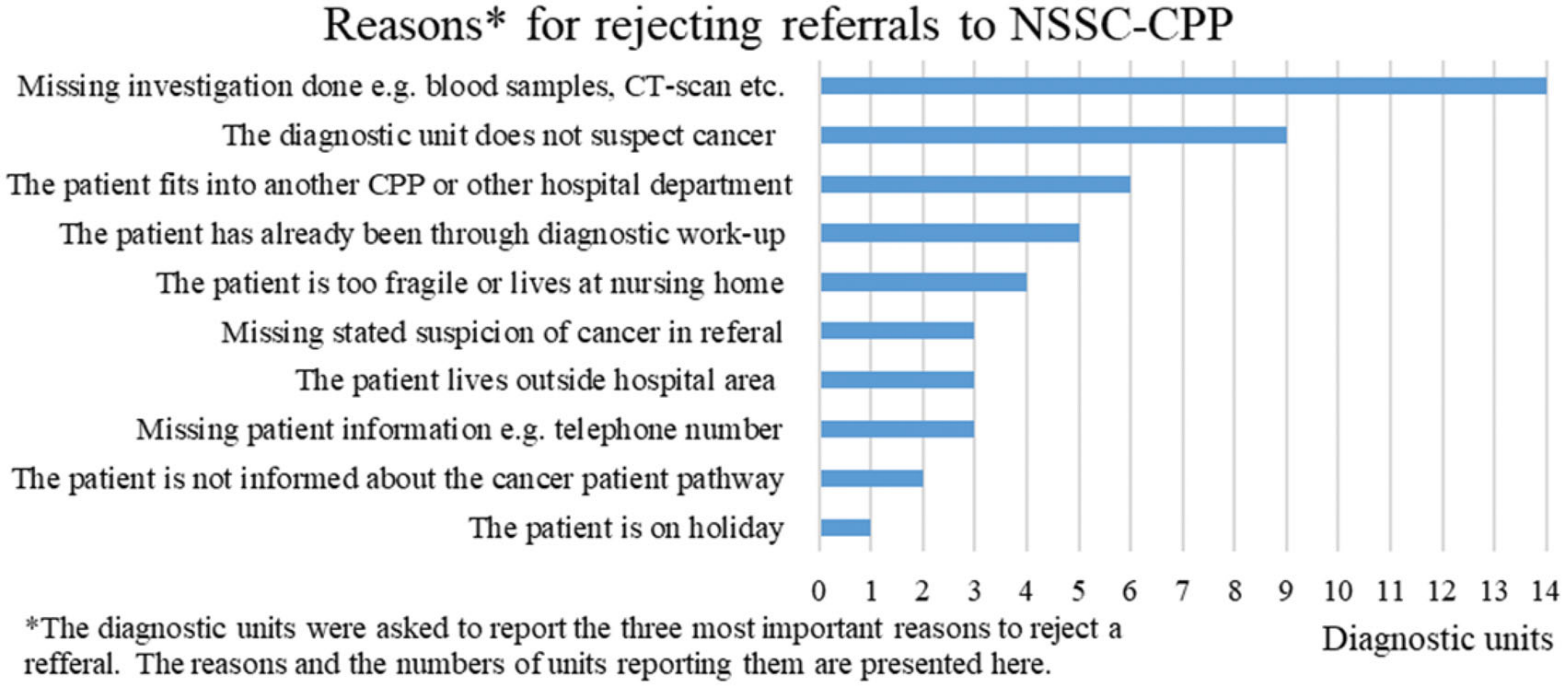
The reported reasons for rejecting referrals to CPP-NSSC and the number of units reporting them.

The overall redirection and rejection rates were 1–15% (mean 6.6%) for redirection, and 0–20% (mean 8.1%) for rejection. Each diagnostic unit was asked to list the three main reasons for rejecting a referral (Figure 1).

### Conferences for discussion of patient plans

All units reported that they had a forum for discussing patient plans, but the type and frequency of forum varied greatly both at the regional and intra-regional levels (Table 2). There was no consensus on which departments that participated in the multidisciplinary team conferences (MDTs).

### Continuation of diagnostic work-up when cancer is not confirmed

Ten diagnostic units reported that they continued the diagnostic work-up, often with the same health professionals, if the patient’s symptoms remained even though the NSSC-CPP had ended with no confirmation of cancer. The other 11 diagnostic units did not have this possibility and discharged patients if the NSSC-CPPs could not confirm cancer.

**Table 2.** Numbers of diagnostic units using the different forums for discussion of patient plans at varying frequencies.

Diagnostic units									Regions
	Never	<1 per month	1 per month	1 per 14 day	1 per week	2–3 per week	>2–3 per week	Every day	Regular conference use^a^
Internal conferences in the unit	4	1	1	1	3	2	1	8	2
Conferences with the radiology department	6	2	1	2	4	0	3	3	1
MDT conferences in the unit	7	2	1	1	5	3	1	1	1
Other departments’ conferences	5	2	3	2	3	5	1	0	1

^a^Number of regions with regular conference use defined as ≥1 per month in all units within the region.

## Discussion

Our results revealed regional and intra-regional differences in the management of the NSSC-CPP in Denmark. In summary, two regions were consistent but had different modalities regarding referrals from GPs. Three regions had intra-regional differences. In these three regions, some diagnostic units had no requirements for general practice and others expected that a CT scan was ordered before referral to the NSSC-CPP.

### Strengths and limitations

A strength of this study was that the diagnostic unit staff were involved from the beginning and contributed to the continuously revision of the questionnaire. Furthermore, the focus group included health professionals from all five health regions in Denmark, which enabled items relevant for the different local organisations to be included in the questionnaire. It would have been interesting with smaller focus groups making room for more interactions and reflections about the questionnaire. However, we experienced a lot of interactions and reflections on the questionnaire as the think-aloud test encouraged the participants to comment on each item, one by one, which made room for discussion. Also, the high level of target group involvement during the entire questionnaire development might have influenced the achieved 100% response rate due to the high content relevance and content coverage of the questionnaire’s subject matter.

A limitation of this study was that only one person from each diagnostic unit completed the questionnaire. It could have been beneficial with more answers from each unit, but as many of the health care professionals only worked part-time in the unit, we were insecure if they all had sufficiently knowledge to answer the questionnaire. Also, it would have been preferable to compare questionnaire responses with medical records to check for discrepancies. Unfortunately, we did not have access to this sort of data.

### Interpretation of results and comparison with other studies

Our results indicated discrepancies between national guidelines and current clinical practice, despite clear guidelines that GPs are responsible for the initial diagnostic work-up, including the initial diagnostic imaging. Several factors might contribute to these discrepancies.

One possible explanation could be that the diagnostic units find it inefficient to ask GPs to order and evaluate blood tests and CT scans if the diagnostic unit has a better set-up themselves. Another factor could be a lack of resources allocated to the diagnostic units. It is striking that some diagnostic units use the same experienced group of medical doctors, while in other units, the staff differed from day-to-day. We cannot tell from our data if this organisation of diagnostic units is chosen to get expertise from different medical specialities, or if it is due to a lack of resources. A third explanation may be that some diagnostic units changed their implementation of guidelines. A diagnostic unit may have implemented multidisciplinary team conferences (MDTs) as recommend by the guidelines, but then evaluated that they were not efficient and un-implemented them. Clearly, more quantitative and qualitative research is needed to understand the discrepancy between guidelines and practice.

Discrepancies can have consequences. Some GPs had the option to refer to two or more different diagnostic units. An recent report indicated that this could be an issue as interviews with GPs revealed that they often felt insecure about the actual requirements [17]. To our knowledge, no other studies have investigated the ways in which the NSSC-CPPs are differently organised from a nationwide perspective. Other published studies in a Danish context have solely focused on one region: the Central Region of Denmark [14,18,21,22] and the Capital Region [19]. One nationwide, population-based cohort study described the characteristics of patients referred to the Danish NSSC-CPP and diagnostic outcomes. [23]. However, this study did not take the different organisation of the pathways into consideration when interpreting the results.

An argument for having GPs perform a filter function, as outlined in the Danish guidelines, is that GPs can order diagnostic tests and perform diagnostic work-up without referring patients to an accelerated CPP, which is a process that can be distressing and strenuous, taxing the mental and physical well-being of some patients [24]. On the other hand, if GPs are not sufficiently supported by radiological descriptions and recommendations, they might be insecure about how to manage this patient group.

The only nationwide study in Denmark [23] found that a CT scan was performed in 41% (range 23–56%) of the completed NSSC-CPPs from 2012 to 2015. Our results indicate that this number might be higher today, as all diagnostic units except one reported that CT scan was the most often used diagnostic imaging method. The total number of CT scans performed in Denmark has increased by 33% from 2013 to 2018 [25]. The rapid rise in CT scans is also a driver for *incidentalomas* [26]. These are incidental imaging findings diagnosed in an asymptomatic patient or a symptomatic patient undergoing imaging for an unrelated reason. Incidentalomas are likely to increase anxiety in the patient and may lead to further investigations and, potentially, overdiagnosis and overtreatment [26,27]. The extent of incidentalomas in the context of NSSC-CPPs has not been investigated but the increase of diagnostic images may increase overdiagnosis and lead to potential delay of diagnosis of other serious diseases.

Despite the different organisation of the NSSC-CPPs in Denmark, they were implemented in Sweden and Norway in 2016 based on the Danish model [28,29]. In the UK, a national NSSC-CPP is not yet implemented, but the Accelerate, Coordinate, Evaluate (ACE) programme is currently evaluating the potential of multidisciplinary diagnostic centres [30]. Neither the implementation of NSSC-CPPs in the Nordic countries, nor the current UK projects, are implemented in such a way that the effects of the different aspects of the modalities can reasonably be evaluated.

In September 2019, the Danish Health Authority initiated a revision of the NSSC-CPP. It is unclear, however, which evidence drives this revision, as the NSSC-CPP is only monitored on the number of completed pathways within the recommended time frames. The Danish NSSC-CPP guideline states that patients should receive equally high-quality care across the country [15]. Quality in healthcare may be divided into effective treatment, patient safety, cost-effectiveness, timeliness, patient centeredness and equality. But it is still not clear which of these dimensions are targeted in the ongoing revision of the NSSC-CPP.

### Implications for research, policy and practice

This study is the first to describe the organisation of the NSSC-CPP both at a regional and intra-regional level in Denmark. This description is central because the Danish GPs meet different requirements when referring to the diagnostic units. This knowledge is important to the Danish Health Authority responsible for monitoring and revising the NSSC-CPP guidance. Still, to improve the diagnostic work-up for patients with non-specific symptoms, the Danish Health Authority needs to combine this knowledge with well-defined quality criteria for the NSSC-CPP. As more countries have adapted the Danish NSSC-CPP, or similar approaches, into the management of their patients with non-specific symptoms of serious disease, our results are an essential contribution as there is a gap regarding the implementation, organisation and effect of the NSSC-CPPs both in Denmark and potential in other countries.

## Conclusion

This study revealed great regional and intra-regional differences in the management of the NSSC-CPP in Denmark. Two regions were consistent but had different modalities regarding referrals from GPs. Three regions had intra-regional differences. Some diagnostic units had no requirements for general practice and others expected that a CT scan was ordered before referral to the NSSC-CPP. Therefore, Danish GPs meet different requirements when referring to the diagnostic units. Great variation was reported in the numbers of rejected referrals with a range of 0–20%. CT scan was the most often used imaging and the forums in which patient pathways were discussed varied in type and frequency. Also, we found differences in how patients were handled when cancer was not confirmed and the NSSC-CPPs ended.

## Supplementary Material

Supplemental Material
