# Supplementary material for: Non-specific symptoms and signs of cancer: different organisations of a cancer patient pathway in Denmark
Source: Scand J Prim Health Care. 2021 Feb 25;39(1):23–30. doi: 10.1080/02813432.2021.1880094 (PMC7971193; doi:10.1080/02813432.2021.1880094)
Supplement: Supplemental Material [file IPRI_A_1880094_SM0266.docx]

Supplementary 1

| **Questionnaire about diagnostic units in Denmark**(ad hoc translation from Danish to English)  This questionnaire is a part of an in-depth investigation of diagnostic units also named diagnostic centres in Denmark. The questionnaire focus on how the diagnostic units are organized and how they manage the referrals to the cancer patient pathway for non-specific symptoms (NSSC-CPP).  In the questionnaire the abbreviation NSSC-CPP will be applied and it is this cancer patient pathway the questionnaire will address.  The questionnaire will be sent electronic to all diagnostic units in Denmark and aims to generate knowledge about the diagnostic units’ modalities including their differences and similarities. You are asked to answer the questions regarding the practice and every day of your diagnostic unit. The questionnaire is a part of a PhD project from The University of Copenhagen and will be published.  The questionnaire is expected to take 10-15 minutes to complete. |
| --- |

On a daily basis, how many health professionals work in your diagnostic unit?
Enter number:

| Secretaries | _____ |
| --- | --- |
| Medical doctors | _____ |
| Nurses | _____ |

Describe the staff’s affiliation to your diagnostic unit. E.g. do they work full time in the diagnostic unit or do they rotate between other departments in the hospital. (describe each health profession)

| Secretaries | ________________________________________ ________________________________________ ________________________________________ |
| --- | --- |
| Medical doctors | ________________________________________ ________________________________________ ________________________________________ ________________________________________ |
| Nurses | ________________________________________ ________________________________________ ________________________________________ ________________________________________ |

In which speciality are the medical doctors in the unit educated?

Tick one or more boxes

❑ Family Medicine

❑ Endocrinology

❑ Gastroenterology

❑ Geriatric

❑ Haematology

❑ Infection medicine

❑ Cardiology

❑ Respiratory

❑ Nephrology

❑ Oncology

❑ Specialist in training (main education)

❑ Specialist in training (introductory education)

❑ Others, enter: _____

**Organisational affiliation.
Which department of the hospital is the diagnostic unit affiliated?**
________________________________________
________________________________________

Physical environment.
Is the diagnostic unit physical located at the same place as it’s department affiliation?

❑ Yes

❑ No

❑ Don’t know

Describe how the physical location of the diagnostic unit and its department affiliation differ:

________________________________________
________________________________________

**Capacity.
In one month, how many new referrals to the NSSC-CPP does the diagnostic unit accept?**

Enter number:

_____

Does your unit manage other patient pathways than patients referred to NSSC-CPP? E.g. patients with cancer of unknown primary site (CUP)

❑ Yes

❑ No

❑ Don’t know

Which other patients than NSSC-CPP does you unit manage?
Tick one or more boxes

❑ Patients with cancer of unknown primary site (CUP)

❑ Patients with non-specific symptoms that do not fit into the NSSC-CPP or other organ specific pathways

❑ Others, enter: __________________________________________________________________________

The following questions address the referrals to NSSC-CPP from the general practice (GP) and referrals from other hospital departments.

Referrals to NSSC-CPP from general practice (GP).
Are there information or investigations that needs to be done before your unit accept a referral?

❑ Yes

❑ No

❑ Don’t know

What is the process if the necessary information or investigations have not been done before referral?
Tick one box

❑ The unit rejects the referral with no recommendation.

❑ The unit rejects the referral and informs the GP about it.

❑ The unit rejects the referral and informs the GP about it. Furthermore, the GP can always contact the diagnostic unit.

❑ The unit does not reject the referral but gather the necessary information and take the tests themselves.

❑ Other, enter: _____

Tick the box for information or investigations that needs to be done before your unit accepts a referral to NSSC-CPP from GP.

Tick one or more boxes

❑ Blood sample

❑ X-ray

❑ Ultrasound

❑ CT scan

❑ Relevant patient information e.g. telephone number

❑ The patient needs to be informed about the initiation of a cancer patient pathway

❑ Other, enter: _____

Referrals to NSSC-CPP from other hospital departments.
Are there information or investigations that needs to be done before your unit accept a referral?

❑ Yes

❑ No

❑ Don’t know

What is the process if the necessary information or investigations have not been done before referral?
Tick one box

❑ The unit rejects the referral with no recommendation.

❑ The unit rejects the referral and informs the hospital department about it.

❑ The unit rejects the referral and informs the hospital department about it. Furthermore, the hospital department can always contact the diagnostic unit.

❑ The unit does not reject the referral but gather the necessary information and take the tests themselves.

❑ Other, enter: _____

Tick the box for information or investigations that needs to be done before your unit accepts a referral to NSSC-CPP from other hospital departments.
Tick one or more boxes

❑ Blood sample

❑ X-ray

❑ Ultrasound

❑ CT scan

❑ Relevant patient information e.g. telephone number

❑ The patient needs to be informed about the initiation of a cancer patient pathway

❑ Other, enter: _____

How many percentages (%) of the received referrals in the unit do you think in average get redirected or rejected?
Enter percentages in numbers:

| Redirected (%) | _____ |
| --- | --- |
| Rejected (%) | _____ |

Enter the three most common reasons to reject a referral to NSSC-CPP:

________________________________________
________________________________________
________________________________________

The following questions concern the diagnostic images the unit use in the diagnostic work-up of patients in the NSSC-CPP.
 
Does the patient’s age influence the choice of CT scan?

❑ Yes

❑ No

❑ Don’t know

Enter the threshold of age for CT scan use:

________________________________________

Which diagnostic image does your unit use in the most NSSC-CPP?

❑ X-ray thorax + ultrasound abdomen

❑ CT thorax and abdomen, if the patient is older than the previous entered threshold for CT scan

❑ Other tests or images, enter _____

Which diagnostic image does your unit use in the most NSSC-CPP?

❑ X-ray thorax + ultrasound abdomen

❑ CT thorax and abdomen

❑ Other tests or images, enter _____

| The following questions concern the forum for discussion of patient pathways in your unit. The word multidisciplinary team conference (MDT-conference) refers to the forum where health professionals from different specialities meet and discuss patient plans. |
| --- |

|  | never | Less often than once a month | Once a month | 1 time every 14 days | Once a week | 2-3 times a week | More often than 2-3 times a week | Every day |
| --- | --- | --- | --- | --- | --- | --- | --- | --- |
| Conferences in own unit | ❑ | ❑ | ❑ | ❑ | ❑ | ❑ | ❑ | ❑ |
| Conferences in own unit including the radiology department | ❑ | ❑ | ❑ | ❑ | ❑ | ❑ | ❑ | ❑ |
| MDT-conferences with two or more specialities represented | ❑ | ❑ | ❑ | ❑ | ❑ | ❑ | ❑ | ❑ |
| Other departments’ conferences | ❑ | ❑ | ❑ | ❑ | ❑ | ❑ | ❑ | ❑ |

Which specialities/departments participate in your MDT-conferences?

________________________________________
________________________________________

When your unit end a NSSC-CPP are you able to continue the diagnostic work-up of the patient even though cancer/ or other serious disease is no longer suspected?

❑ Yes

❑ No

❑ Don’t know

**Describe how your unit manage the diagnostic work-up of patients when the NSSC-CPP is ended:**

________________________________________
________________________________________
________________________________________

When ending a NSSC-CPP when cancer is no longer suspected. Which hospital departments do you refer patients to?

Write the three departments most often referred to:

________________________________________
________________________________________
________________________________________

How many calendar days varies a NSSC-CPP in average in your unit? (from referral to the NSSC-CPP is ended)

**Enter number:**

_____

If you think there are any important issues which we have not asked you in this questionnaire, feel free to write all your comments or questions here:

________________________________________
________________________________________
________________________________________
________________________________________
________________________________________

| **Thank you for answers!** |
| --- |
